# From bedside to bench to clinic trials: identifying new treatments for severe asthma

**DOI:** 10.1242/dmm.012070

**Published:** 2013-07

**Authors:** Amarjit Mishra, Xianglan Yao, Stewart J. Levine

**Affiliations:** 1 Cardiovascular and Pulmonary Branch, National Heart, Lung and Blood Institute, National Institutes of Health, Bethesda, MD 20892-1590, USA

## Abstract

Asthmatics with a severe form of the disease are frequently refractory to standard medications such as inhaled corticosteroids, underlining the need for new treatments to prevent the occurrence of potentially life-threatening episodes. A major obstacle in the development of new treatments for severe asthma is the heterogeneous pathogenesis of the disease, which involves multiple mechanisms and cell types. Furthermore, new therapies might need to be targeted to subgroups of patients whose disease pathogenesis is mediated by a specific pathway. One approach to solving the challenge of developing new treatments for severe asthma is to use experimental mouse models of asthma to address clinically relevant questions regarding disease pathogenesis. The mechanistic insights gained from mouse studies can be translated back to the clinic as potential treatment approaches that require evaluation in clinical trials to validate their effectiveness and safety in human subjects. Here, we will review how mouse models have advanced our understanding of severe asthma pathogenesis. Mouse studies have helped us to uncover the underlying inflammatory mechanisms (mediated by multiple immune cell types that produce Th1, Th2 or Th17 cytokines) and non-inflammatory pathways, in addition to shedding light on asthma that is associated with obesity or steroid unresponsiveness. We propose that the strategy of using mouse models to address clinically relevant questions remains an attractive and productive research approach for identifying mechanistic pathways that can be developed into novel treatments for severe asthma.

## Bedside: a clinical perspective on severe asthma

Asthma is a common, chronic inflammatory disease of the airways that affects over 300 million individuals worldwide and is associated with 250,000 premature deaths each year (Bousquet and Khaltaev, 2007; Bousquet et al., 2010). Severe asthma has recently been defined by the World Health Organization as ‘uncontrolled asthma which can result in risk of frequent severe exacerbations (or death) and/or adverse reactions to medications and/or chronic morbidity (including impaired lung function or reduced lung growth in children)’ (Bousquet et al., 2010). In addition, a recent statement from the American Thoracic Society and the European Respiratory Society defined severe asthma as being difficult to control with treatment after excluding ‘modifiable factors, such as poor adherence, smoking and comorbidities’ (Reddel et al., 2009). Compared with individuals with mild disease, severe asthmatics often have late disease onset, decreased atopy, elevated sputum neutrophilia and impaired pulmonary function (Anderson, 2008; Wenzel, 2012a) (see Box 1 for a glossary of clinical terms). Unsurprisingly, morbidity is higher in severe asthmatics than in those with milder disease, as indicated by an increase in emergency health care visits, hospitalizations and intensive care unit (ICU) utilization in such individuals (Moore et al., 2007). Therefore, severe asthma is an important public health problem. Treatment of severe asthma typically consists of high doses of inhaled corticosteroids, often in combination with an inhaled long-acting β_2_-agonist and other controller medications, such as leukotriene modifiers (e.g. leukotriene receptor antagonists or 5-lipoxygenase inhibitors). Only a limited number of adjunctive treatment options are currently available for severe asthmatics whose symptoms are not adequately controlled by standard therapies. These alternative treatments include oral corticosteroids, which have substantial side effects; omalizumab, a parenterally administered monoclonal antibody directed against IgE that can be effective in individuals with IgE-mediated allergic asthma; and bronchial thermoplasty, a recently introduced approach that requires several invasive bronchoscopic procedures.

Box 1. **Clinical terms**

**Airflow obstruction:** reduction in the amount of gas that can be exhaled from the lung, due to airway narrowing.

**Airway hyperresponsiveness (AHR):** the enhanced contractility of airway smooth muscle in response to a bronchoconstricting stimulus.

**Airway remodeling:** pathogenic changes that increase airway wall thickness and decrease airway luminal diameter, such as mucous cell metaplasia and mucus hypersecretion, hypertrophy and hyperplasia of airway epithelial cells and smooth muscle cells, and deposition of collagen and extracellular matrix proteins.

**Asthma phenotypes:** subgroups of asthmatic patients characterized by distinct clinical or pathogenic features.

**Atopy:** a genetic predisposition to develop type I hypersensitivity reactions to antigens that result in allergic diseases, such as asthma, allergic rhinitis or atopic dermatitis.

**Bronchial thermoplasty:** a recently introduced treatment for asthma that uses several bronchoscopic procedures to thermally ablate airway smooth muscle.

**Bronchoalveolar lavage (BAL):** a bronchoscopic procedure where a videobronchoscope is inserted into the airway and saline instillations are recovered for subsequent analysis.

**FEV_1_:** forced expiratory volume in 1 second; a pulmonary function test that is used to measure airway obstruction in asthma.

**Methacholine challenge:** inhalation of aerosolized methacholine, a cholinergic agonist, can be used to induce bronchoconstriction as a diagnostic test for airway hyperresponsiveness in asthma.

**Neutrophilia:** an increase in neutrophils (polymorphonuclear leukocytes).

**Omalizumab:** a humanized monoclonal antibody that binds IgE.

**Pauci-granulocytic:** the absence of increased numbers of eosinophils or neutrophils in sputum samples from a subset of severe asthmatics.

**Spirometry:** a clinical test to quantify airflow obstruction during an exhalation maneuver by measuring FEV_1_ (see above).

**Sputum:** liquid that is ejected from the lungs and airways via the mouth.

It is increasingly recognized that severe asthma is not a single disease process, but instead is comprised of several clinical or inflammatory subgroups or phenotypes that probably reflect distinct underlying mechanistic pathways (Wenzel, 2012b). For example, two cluster analyses have identified several severe asthma clinical phenotypes characterized by either early or late onset of disease, presence or absence of atopy or obesity, female or male gender, and predominance of sputum eosinophils with or without neutrophils (Haldar et al., 2008; Moore et al., 2010). Eosinophils are key effector inflammatory cells in allergic asthma that are characteristically recruited to the lung in the setting of adaptive immune responses mediated by Th2-type CD4^+^ T cells, whereas neutrophilic airway inflammation has been associated with corticosteroid treatment in severe asthma and can be mediated by Th1- or Th17-type T cells (see Box 2) (Barnes, 2008; Holgate, 2012; Kim et al., 2010; Wenzel, 2012a). Sputum analyses from severe asthmatics have also identified a pauci-granulocytic inflammatory phenotype that does not have increased numbers of inflammatory cells (such as sputum eosinophils or neutrophils), which suggests the involvement of non-inflammatory mechanisms mediated by airway remodeling responses that lead to excessive airway narrowing (Grainge et al., 2011; Simpson et al., 2006). The identification of distinct severe asthma phenotypes has fostered the concept of specific targeted or personalized therapies. For example, omalizumab has entered clinical practice for the treatment of moderate to severe persistent asthma in individuals with documented allergy to a perennial aeroallergen, and with serum IgE levels between 30 and 700 IU/ml (Fanta, 2009). Thus, there is a growing need to develop additional therapies for the management of severe asthma.

Box 2. **Inflammatory and immune cells in asthma**

**Dendritic cell:** immune cells that induce adaptive immune responses in allergic asthma by presenting antigens to T cells.

**Eosinophil:** granular leukocytes that are recruited to the lung by IL-5 and C-C chemokines (CCL11, CCL24 and CCL26) to mediate allergen-induced airway inflammation.

**Innate lymphoid cells (ILCs):** innate effector leukocytes that respond to IL-25 and IL-33 and mediate type 2 immune responses by secreting IL-5 and IL-13. These cells have also been termed nuocytes, natural helper cells or innate helper 2 cells.

**Invariant NKT (iNKT) cells:** an NKT (see below) cell subset that expresses the invariant T cell receptor that recognizes glycolipid antigens.

**Mast cell:** resident tissue cells with basophilic intracytoplasmic granules that release histamine upon binding of IgE to mediate allergic inflammatory responses.

**Natural killer T (NKT) cells:** a subset of CD1d-restricted T cells that display characteristics of both T cells and natural killer (NK) cells.

**Neutrophil:** granular leukocytes that facilitate host defense against bacterial and fungal pathogens, but also mediate airway inflammation in severe asthma.

**Th1 T cells:** CD4^+^ T cells that express T-helper 1 (Th1)-type cytokines, such as interferon-γ (IFNγ), and facilitate cell-mediated immune responses against viral and bacterial infections.

**Th2 T cells:** CD4^+^ T cells that express T-helper 2 (Th2)-type cytokines, such as interleukin (IL)-4, IL-5 and IL-13, and mediate immune responses in allergic diseases and parasitic infections.

**Th17 T cells:** CD4^+^ T cells and γδ T cells that express T-helper 17 (Th17)-type cytokines, such as IL-17A, and mediate immune responses against bacterial and fungal infections, as well as autoimmunity.

**Type 2 myeloid (T2M) cells:** a recently identified, steroid-resistant, bone-marrow-derived cell population that is recruited to asthmatic airways in response to allergen challenge and mediates type 2 airway inflammation independently of CD4^+^ Th2 cells.

## Bedside to bench: using mouse models to identify new treatment approaches for severe asthma

One approach to solving the challenge of developing new treatments for severe asthma is to use experimental mouse asthma models to improve our understanding of the processes implicated in disease pathogenesis, which include airway inflammation, airway remodeling and airway hyperresponsiveness (AHR). The insights gained from these mouse studies can then be translated back to the clinic as new treatment approaches, subject to evaluation in clinical trials to validate their effectiveness and safety in human subjects. Here, we will review how research utilizing mouse asthma models has generated clinically relevant findings regarding the pathogenesis of severe asthma and has revealed targets to exploit in the development of new treatments.

### Mouse models of steroid-unresponsive asthma

A hallmark of severe asthma is refractoriness to treatment with high doses of inhaled or oral corticosteroids. As summarized in Table 1, the ability of steroids to suppress airway inflammation, airway remodeling and AHR has been investigated in several mouse models of allergic asthma induced by administration of either ovalbumin (OVA) or house dust mite (HDM), as well as in experimental asthma induced by administration of the Th2 cytokine IL-13, either as an exogenous recombinant protein or as an IL-13-expressing adenovirus (Birrell et al., 2003; Essilfie et al., 2012; Ito et al., 2008; Kibe et al., 2003; Therien et al., 2008; Yao et al., 2010). Several interesting observations regarding steroid unresponsiveness in asthma were made during these studies. First, in mouse models of OVA- and HDM-induced allergic asthma, eosinophilic and lymphocytic airway inflammatory responses are steroid-responsive, whereas the ability of steroids to suppress neutrophilic airway inflammation varies. Second, mouse asthma models induced by the Th2 cytokine IL-13 are associated with steroid-responsive eosinophilic and lymphocytic airway inflammation, which are dissociated from steroid-resistant neutrophilic airway inflammation, mucus production and AHR (Kibe et al., 2003; Therien et al., 2008). Third, the corticosteroid dose required to attenuate AHR in mouse models is higher than that needed to reduce eosinophilic airway inflammation or invoke small, albeit statistically significant, decreases in AHR (Birrell et al., 2003; Yao et al., 2010). Fourth, respiratory infections, which are a common trigger for asthma exacerbations, can contribute to steroid unresponsiveness in asthma: in a mouse model of OVA-induced allergic airways disease, concomitant lung infection with a non-typeable *Haemophilus influenza* was associated with increases in Th17-mediated neutrophilic airway inflammation and reductions in eosinophilic inflammation (Essilfie et al., 2011). Furthermore, combined *H. influenza* infection and OVA-induced allergic airways disease results in steroid-unresponsive neutrophilic airway inflammation and AHR, whereas these manifestations are steroid-responsive in noninfected OVA-challenged mice (Essilfie et al., 2012). This suggests that co-existing *H. influenza* infection and allergic airways disease leads to chronic infection that facilitates the development of steroid-resistant, Th17-mediated, neutrophil-predominant asthma. These findings support the concept that treating bacterial infections in steroid-resistant asthmatics might be beneficial.

**Table 1.**
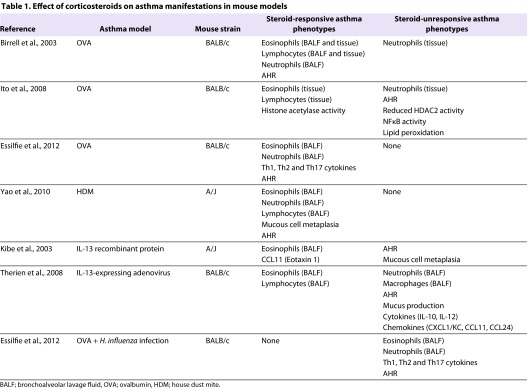
Effect of corticosteroids on asthma manifestations in mouse models

Histone deacetylases (HDACs) play an important role in reducing or silencing pro-inflammatory gene transcription, and have been implicated in the pathogenesis of steroid-unresponsive asthma. HDAC2 has been shown to deacetylate the glucocorticoid receptor, which then binds to NFκB and attenuates the expression of pro-inflammatory genes (Barnes, 2011; Ito et al., 2008). In addition, reduced HDAC2 activity in peripheral blood mononuclear cells from severe asthmatics has been shown to correlate with impaired sensitivity to steroid treatment (Hew et al., 2006). Studies analyzing bronchoalveolar lavage (BAL) cells and bronchial biopsies have generated discordant results as to whether HDAC expression is modified in severe asthmatic individuals (Butler et al., 2012; Li et al., 2010b). Analysis of mouse models, however, has supported a role for HDACs in steroid-resistant severe asthma. For example, reduced HDAC activity has been linked with steroid-resistant neutrophilic airway inflammation in a mouse OVA model simulating an acute asthma exacerbation (Ito et al., 2008). In this model, steroids did not suppress neutrophilic inflammation and AHR. Furthermore, dexamethasone, a potent synthetic corticosteroid with anti-inflammatory and immunosuppressive properties, did not reverse OVA-induced decreases in HDAC2 protein or increases in NFκB activity (Ito et al., 2008). Increases in lipid peroxidation were also unresponsive to steroid treatment, suggesting a role for oxidative stress in the reduced HDAC2 activity. Collectively, these findings support impaired nuclear recruitment of HDAC2 as a potential mechanism of steroid-resistant neutrophilic airway inflammation in asthma. Similarly, OVA-challenged mice with a conditional deletion of HDAC1 in CD4^+^ T cells display a phenotype of increased airway eosinophilia and Th2 cytokine production, mucus hypersecretion and AHR (Grausenburger et al., 2010). Thus, therapeutic strategies aimed at enhancing HDAC activity might be beneficial in steroid-unresponsive asthma.

**Case study**

The real-world challenges facing severe asthmatics are illustrated by the following case study of a middle-aged female patient with a history of allergic asthma since childhood. She has had severe disease for the past two decades, as evidenced by yearly asthma decompensations that require an urgent care visit, as well as multiple asthma hospitalizations, one of which was associated with respiratory failure requiring ICU admission. The patient’s asthma was inadequately controlled despite multiple medications, which included high doses of an inhaled corticosteroid plus a long-acting β2-agonist, and an oral leukotriene receptor antagonist. In addition, she required treatment with oral prednisone (10 mg daily), which resulted in several corticosteroid-related toxicities including weight gain, cushingoid features (moon-shaped facies, buffalo hump and truncal obesity) and osteopenia (reduction in bone mass). To circumvent these side effects, specifically osteopenia, the patient required treatment with alendronate (a bisphosphonate medication used to prevent and treat loss of bone mass), calcium and vitamin D. Her serum immunoglobulin E was mildly elevated. Spirometry revealed severe airflow obstruction on repeated determinations with intermittent positive bronchodilator responses to an inhaled β2-agonist. Thus, at time of presentation, the patient had poorly controlled asthma despite daily treatment with oral corticosteroids that were causing substantial side effects. Induced sputum analysis revealed >7% eosinophils, consistent with an eosinophil-predominant inflammatory phenotype that might in the future be amenable to treatment with an anti-IL-5 therapy. A therapeutic trial of omalizumab could also be considered. This case clearly illustrates that there remains an unmet need for the development of new treatment options for severe asthmatics that are safe, efficacious, easy to administer and affordable. Furthermore, therapies for severe asthma might be more effective if they are personalized to target the pathways that mediate the clinical phenotype of each patient.

A mouse model of HDM-induced asthma has also been used to identify steroid-unresponsive genes that participate in the pathogenesis of asthma and could thereby represent potential new targets for therapeutic interventions (Yao et al., 2011b). Genome-wide analysis of the lung transcriptome in HDM-challenged mice showed that apolipoprotein E expression increases in response to HDM, and expression remains persistently high even after treatment with dexamethasone. Furthermore, in apolipoprotein-E-deficient mice, HDM challenge results in augmentation of AHR and mucous cell metaplasia (Yao et al., 2010). Together, these data indicate that endogenous apolipoprotein E has a protective role in asthma. Apolipoprotein E, which is expressed by alveolar macrophages, mediates this protective effect by binding to low density lipoprotein (LDL) receptors on airway epithelial cells. Administration of an apolipoprotein E mimetic peptide, corresponding to the LDL-receptor-binding domain of the holo-apolipoprotein E, not only reverses the increases in HDM-induced AHR and mucous cell metaplasia, but also attenuates eosinophilic airway inflammation and the production of Th2 and Th17 cytokines in an asthma model. This suggests that administration of apolipoprotein E mimetic peptides might represent a novel treatment approach for individuals with steroid-unresponsive severe asthma. Interestingly, recent studies have identified a potential link between apolipoprotein E and protein phosphatase 2A (PP2A) in the treatment of severe asthma. Upon internalization into cells, apolipoprotein E (or apolipoprotein E mimetic peptides) bind SET, a physiological inhibitor of PP2A, which liberates PP2A to attenuate pro-inflammatory signaling (Christensen et al., 2011). This could be beneficial from a treatment perspective, because PP2A expression and activity are reduced in peripheral blood mononuclear cells from severe asthmatics (Kobayashi et al., 2011).

### Type 2 immune-mediated mechanisms in severe asthma pathogenesis

**Fig. 1.**
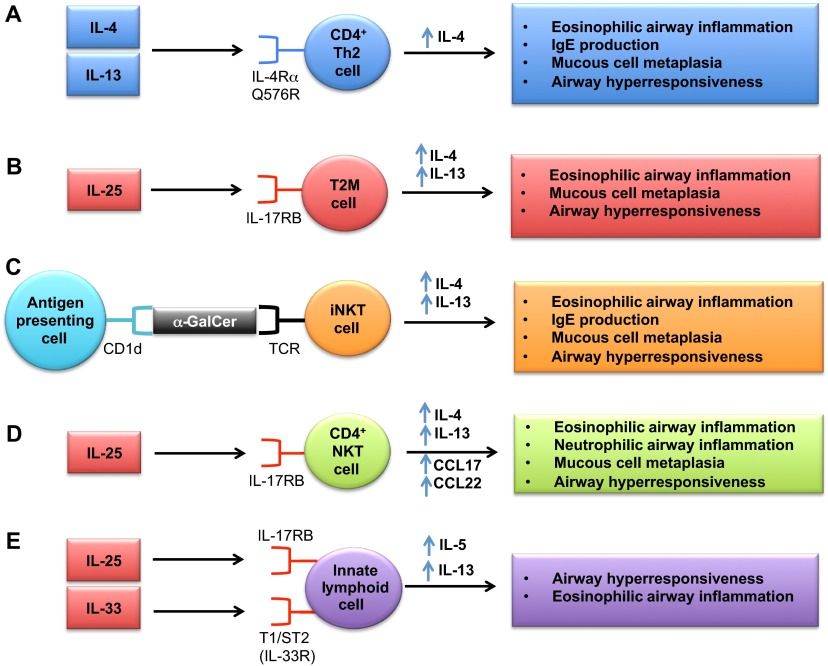
**Type 2 immune pathways in mouse models of severe asthma.** (A) CD4^+^ T cells mediate type 2 immune responses by producing canonical cytokines, such as IL-4, IL-5 and IL-13. Knock-in mice expressing the Q576R polymorphism of the human IL-4 receptor α chain (IL-4Rα) recapitulate a severe asthma phenotype seen in African-American patients manifested by increased IL-4 production by CD4^+^ Th2 lymphocytes. Recent mouse studies have also identified that non-CD4^+^ cells play an important role in mediating type 2 immune responses in severe asthma. These include: (B) type 2 myeloid (T2M) cells, which are a steroid-resistant cell population in asthmatic airways that produce IL-4 and IL-13 in response to IL-25, (C) invariant natural killer T (iNKT) cells that produce IL-4 and IL-13 in response to the glycolipid α-galactosylceramide (α-GalCer) when presented by the MHC class I-like protein, CD1d, (D) CD4^+^ NKT cells that express IL-17RB and produce IL-13, IL-4 and Th2 chemokines in response to IL-25, and (E) innate effector lymphoid cells (ILCs) that produce IL-5 and IL-13 in response to IL-25 or IL-33.

Type 2 immune responses mediated by a canonical set of Th2 cytokines, which include IL-4, IL-5 and IL-13 and are produced by Th2-type CD4^+^ T cells, play a central role in the pathogenesis of allergic asthma (Barnes, 2008; Holgate, 2012; Kim et al., 2010; Wenzel, 2012b). For example, IL-4 regulates the differentiation of naive Th0 cells to Th2 cells, IL-4 and IL-13 mediate isotype class switching of B cells to IgE production, IL-5 promotes eosinophil differentiation and survival, and IL-13 induces mucus hypersecretion, AHR and chemokine production. Mouse models have provided important insights into the role of type 2 immune responses in the pathogenesis of severe asthma. In one study, a knock-in mouse model of the human Q576R variant of the interleukin-4 receptor α chain (IL-4Rα) was used to investigate the relationship between this genotype and a phenotype of severe asthma and low lung function that is more common in African-Americans compared with Caucasians (Tachdjian et al., 2009). OVA-challenged mice expressing the IL-4Rα R576 mutant allele had enhanced eosinophilic airway inflammation, mucous cell metaplasia and airway remodeling compared with mice expressing the conserved Q576 wild-type allele (Fig. 1A). The polymorphism was also associated with increases in IL-4 production by Th2 cells, antigen-specific IgE production and expression of IL-13-responsive genes. Similarly, intranasal administration of IL-13 to mice with the Q576R substitution leads to enhanced AHR, airway eosinophilia and mucous cell metaplasia (Tachdjian et al., 2009). These findings confirmed a causal relationship between the Q576R IL-4Rα polymorphism and the clinical phenotype of heightened asthma prevalence and severity in African-Americans due to enhanced Th2 inflammation.

It has recently been recognized that type 2 immune responses are not only generated by CD4^+^ Th2 T cells, but are also produced by other cell types, such as invariant natural killer T (iNKT) cells, type 2 myeloid (T2M) cells and innate lymphoid cells (Holgate, 2012; Kim et al., 2010). Furthermore, recent studies using mouse models have provided evidence that non-CD4^+^ cells play an important role in mediating type 2 immune responses in steroid-resistant asthma (Petersen et al., 2012). A mouse model of cockroach-induced chronic airway inflammation identified T2M cells as a steroid-resistant cell population in asthmatic airways that mediates type 2 airway inflammation independently of CD4^+^ Th2 cells (Fig. 1B) (Petersen et al., 2012). Allergen-exposed mice demonstrate increased expression of the IL-17 family member IL-25 (also known as IL-17E), which induces Th2 cytokine production by T2M cells via the IL-25 receptor, IL-17RB, as well as by CD4^+^ T cells. Consistent with this, IL-17RB knockout mice show decreases in eosinophilic lung inflammation and mucous cell metaplasia following cockroach antigen challenge, and the adoptive transfer of T2M cells successfully reconstitutes IL-25-mediated responses. In contrast, dexamethasone treatment did not reduce the IL-25-induced T2M pulmonary responses characterized by increases in type 2 cytokines, airway inflammation, mucous cell metaplasia and AHR. Lastly, the relevance of T2M cells to human asthma was suggested by a significant increase in the number of CD11b^+^CD16^+^CD177^+^ granulocytic cells that express 17RB^+^ and produce IL-4 and IL-13 in response to IL-25 stimulation, in peripheral blood samples from asthmatic subjects. Thus, in response to IL-25, IL-17RB^+^ T2M cells might be recruited to the lung and mediate steroid-unresponsiveness in asthma.

iNKT cells that produce IL-4 and IL-13 represent another cell type that promotes AHR independently of CD4^+^ Th2 cells (Fig. 1C) (Meyer et al., 2006). iNKT cells express a highly restricted T-cell-receptor repertoire consisting of Vα14-Jα18 in mice and Vα24-Jα18 in humans that selectively recognizes the glycolipid α-galactosylceramide (α-GalCer) when presented by the major histocompatibility complex (MHC) class I-like protein, CD1d (Holgate, 2012; Kim et al., 2010; Meyer et al., 2006). It has been reported that the percentage of iNKT cells in the lungs of severe asthmatics is higher than in non-asthmatics or those with well-controlled asthma, which suggests a role in severe disease; however, some controversy exists regarding the role of iNKT cells in human disease (Kim et al., 2010; Matangkasombut et al., 2009). MHC-II-deficient mice that lack conventional CD4^+^ T cells, but have iNKT cells, display increased AHR at baseline and following challenge with α-GalCer (Meyer et al., 2006). Activation of iNKT cells also increases serum levels of IL-4 and IgE, in addition to increasing eosinophilic and lymphocytic peri-bronchiolar inflammation. iNKT cells have also been implicated in the pathogenesis of AHR following allergen challenge and ozone exposure (Kim et al., 2010). Similarly, an NKT-cell–macrophage immune axis has been identified that mediates chronic airway disease in a mouse model of Sendai virus infection (Kim et al., 2008). In this model, viral infection activates CD4^−^ iNKT cells via glycolipid-loaded CD1d, which then induces IL-13 production by lung macrophages and subsequent mucous cell metaplasia. In addition, a subset of IL-17RB^+^ CD4^+^ NKT cells has been identified, which expresses IL-13 and, to a lesser extent, IL-4, as well as Th2 chemokines (CCL17 and CCL22), upon stimulation by IL-25 *in vitro*. These cells mediate OVA/IL-25-dependent AHR, mucous cell metaplasia and airway inflammation *in vivo* (Fig. 1D) (Terashima et al., 2008).

Innate lymphoid cells (ILCs; which have also been termed nuocytes, natural helper cells or innate helper 2 cells) are innate effector lymphoid cells that respond to IL-25 and IL-33 to mediate type 2 immune responses independently of CD4^+^ Th2 T cells or B cells, via the secretion of IL-5 and IL-13, but little IL-4 (Barlow et al., 2012; Chang et al., 2011; Spits et al., 2013). ILCs that produce IL-13 in response to IL-33 secreted by alveolar macrophages were initially found to mediate influenza-induced AHR in mice (Chang et al., 2011). ILCs have also been shown to be a major innate source of IL-13 in allergen-challenged lungs, and to mediate IL-25-induced AHR and eosinophilia in mouse asthma (Fig. 1E) (Barlow et al., 2012). Similarly, in a mouse asthma model, NKT cells activated by glycolipid antigens (α-GalCer) directly induce alveolar macrophages and dendritic cells to produce IL-33, which activates NKT cells and ILCs to secrete IL-13 and thereby induce AHR and airway inflammation (Kim et al., 2012). Furthermore, IL-33 is upregulated in airway smooth muscle from asthmatics, and dexamethasone does not attenuate TNF-induced IL-33 upregulation, which suggests a role for IL-33 in severe asthma (Préfontaine et al., 2009).

Studies of experimental asthma models have suggested a role for Lyn in the development of a type 2 immune phenotype in severe asthma. Lyn is a Src family non-receptor tyrosine kinase that is expressed by hematopoietic cells (with the exception of T cells) infiltrating the inflamed asthmatic airway (Beavitt et al., 2005). OVA-challenged Lyn-deficient mice display a phenotype of severe, persistent airway inflammation, concomitant with an increase in bronchoalveolar lavage fluid (BALF) eosinophils, macrophages and CD4+ T cells, as well as an increase in Th2 cytokines (IL-4 and IL-5), mast cell degranulation, AHR, IgE production and Th2-polarizing dendritic cells. This demonstrates that Lyn functions as a negative regulator of severe type-2-mediated airway inflammation via a non-T-cell-mediated mechanism. Mice deficient in Nrf2 (nuclear erythroid 2 p45-related factor 2), a redox-sensitive basic leucine zipper transcription factor that mediates the transcription of numerous antioxidant genes, also have a phenotype of severe OVA-induced eosinophilic airway inflammation, increased expression of Th2 cytokines, mucous cell metaplasia, and AHR that occurs secondary to the attenuated transcription of multiple antioxidant genes (Rangasamy et al., 2005). This suggests that Nrf2-dependent antioxidant pathways could be impaired in severe asthma.

### IL-17-dependent, steroid-unresponsive neutrophilic airway inflammation in severe asthma

Neutrophils are a predominant cell type found in airway secretions during severe asthma exacerbations and have been associated with chronic airway narrowing in asthma (Fahy, 2009). In addition, some severe asthmatics can be phenotypically characterized by sputum neutrophilia, together with adult-onset disease, severe airflow obstruction, systemic corticosteroid use and high healthcare utilization (Wenzel, 2012b). Furthermore, steroids can enhance airway neutrophilia by inhibiting neutrophil apoptosis and by promoting neutrophil activation (Wenzel, 2012b). Thus, in a subset of patients, neutrophils play an important role in the pathogenesis of severe asthma. IL-17A, which is primarily produced by CD4^+^ Th17 cells, as well as by γδ T cells, natural killer (NK) cells, mast cells and neutrophils, is increased in the lungs of severe asthmatics and correlates with sputum neutrophilia (Miossec and Kolls, 2012). Mechanisms by which IL-17 promotes neutrophilic inflammation include enhanced granulopoiesis, chemotaxis and survival, which are mediated via CXCL8 (IL-8) and granulocyte-colony stimulating factor (Miossec and Kolls, 2012).

**Fig. 2.**
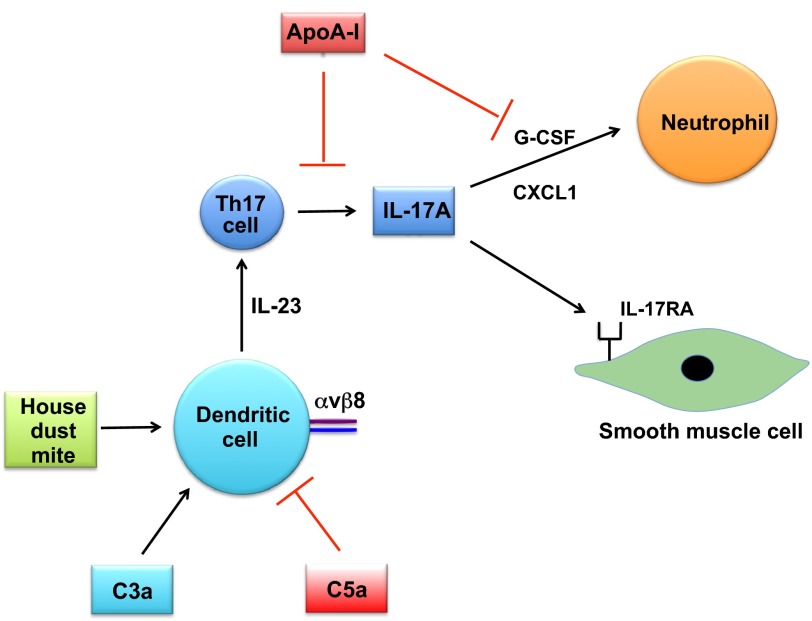
**Th17 immune pathways in mouse models of severe asthma.** IL-17 production by Th17 cells in mouse models mediates steroid-unresponsive neutrophilic airway inflammation and airway hyperresponsiveness (AHR). In a house dust mite (HDM) model of severe asthma, complement factor C3a enhances, whereas C5a protects against, dendritic-cell-mediated IL-23 production, Th17 responses and AHR. Mice that lack the αvβ8 integrin on dendritic cells are also protected from AHR owing to a loss of Th17 cells. Furthermore, IL-17A has been shown to bind directly to IL-17 receptor A on airway smooth muscle cells, which enhances contractile force generation. In mouse models, endogenous apolipoprotein A-I reduces ovalbumin (OVA)-induced neutrophilia by attenuating production of G-CSF and IL-17.

Mouse-model-based studies of Th17 cells and IL-17 have provided key insights into the role of the IL-17 pathway in the pathogenesis of severe asthma (Fig. 2). For example, the adoptive transfer of *in vitro* polarized, antigen-specific Th17 cells to OVA-challenged mice induces BALF neutrophilia and AHR that is resistant to steroids and is associated with increased levels of CXCL1 (GROα, KC) and G-CSF in BALF, whereas the adoptive transfer of *in vitro* polarized antigen-specific Th2 cells induces BALF eosinophila and lymphocytosis that is steroid-responsive (McKinley et al., 2008). This supports the concept that Th17 cells mediate steroid-unresponsive neutrophilic airway inflammation and AHR in severe asthma. A mouse model of HDM-induced asthma has also shown that IL-17A-induced increases in AHR are regulated by the complement system. In particular, C3a production induces, whereas C5a inhibits, dendritic-cell-derived IL-23 production, which in turn upregulates IL-17A production by Th17 cells and thus leads to increases in AHR (Lajoie et al., 2010). Furthermore, neutralization of IL-17A significantly attenuates HDM-induced BALF neutrophilia. This work provided a link between complement activation and the IL-17 pathway in asthma. Additional evidence regarding the role of IL-17A in AHR has come from conditional knockout mice that lack the αvβ8 integrin on dendritic cells (Kudo et al., 2012). These mice do not generate CD4^+^ αβ Th17 cells and are protected from allergen-induced AHR. Furthermore, IL-17A binds to IL-17 receptor A on airway smooth muscle cells and directly enhances contractile force generation of mouse tracheal rings (Kudo et al., 2012). Collectively, these studies suggest that therapies targeting IL-17A might be beneficial for the treatment of steroid-unresponsive neutrophilic airway inflammation and AHR. Consistent with this, an anti-IL-17 neutralizing antibody has entered human clinical trials for asthma (Miossec and Kolls, 2012).

Interestingly, apolipoprotein A-I has been shown to negatively regulate neutrophilic airway inflammation in allergen-induced mouse models (Fig. 2). Apolipoprotein A-I (apoA-I) is the major protein component of high-density-lipoprotein particles and mediates reverse cholesterol transport out of cells by interacting with the ATP-binding cassette (ABC) transporter A1 (ABCA1) (Navab et al., 2011; Osei-Hwedieh et al., 2011). ApoA-I-deficient (*apoA-I*^−/−^) mice that are challenged with OVA demonstrate augmented BALF neutrophilia that is secondary to an increase in G-CSF production (Dai et al., 2012). The phenotype of augmented BALF neutrophilia can be reversed by administration of an apoA-I mimetic peptide, 5A. Similarly, administration of the 5A apoA-I mimetic peptide to HDM-challenged wild-type A/J mice attenuates increases in IL-17A expression, BALF neutrophilia and AHR, as well as increases in mucous cell metaplasia and numbers of BALF eosinophils and lymphocytes (Yao et al., 2011a). Taken together, these studies imply that administration of apoA-I mimetic peptides might be beneficial for the treatment of neutrophil-predominant severe asthma phenotypes.

### Th1-dependent neutrophilic airway inflammation and AHR in severe asthma

**Fig. 3.**
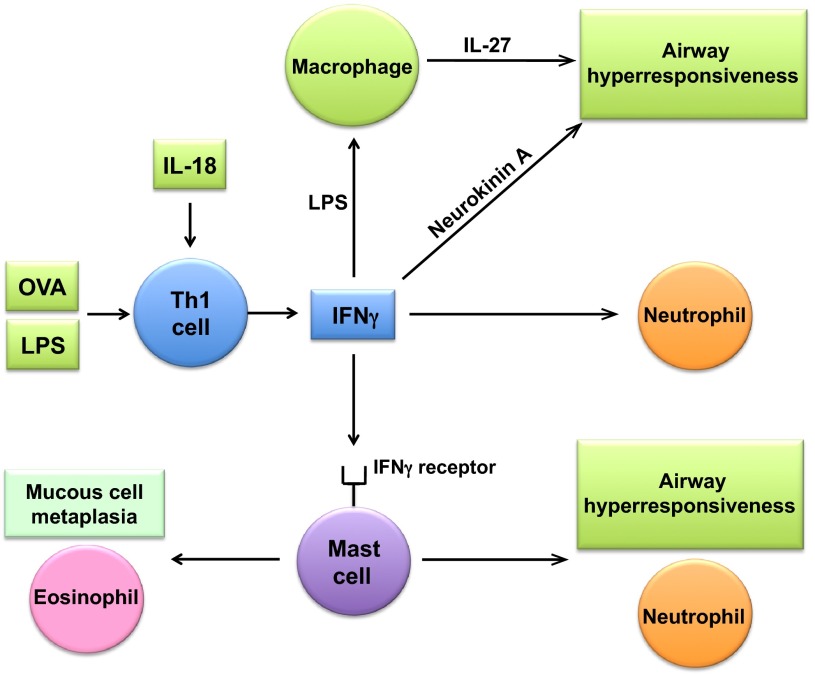
**Th1 immune pathways in mouse models of severe asthma.** IFNγ-dependent Th1 responses mediate the pathogenesis of neutrophilic airway inflammation and airway hyperresponsiveness (AHR) in mouse models of severe asthma. The adoptive transfer of ovalbumin (OVA)-specific IFNγ-producing Th1 cells plus lipopolysaccharide (LPS) induces steroid-resistant AHR and neutrophilia. IFNγ-producing Th1 cells are activated in response to antigen (OVA) and IL-18. Mouse model studies have shown that IFNγ-dependent AHR in severe asthma involves IL-27 production by macrophages and the binding of neurokinin A to neurokinin-2 receptors on airway smooth muscle cells. IFNγ can also activate mast cell IFNγ receptors to promote AHR, mucous cell metaplasia, and neutrophilic and eosinophilic airway inflammation.

Both human and mouse studies have provided evidence suggesting a role for Th1 cells in the pathogenesis of severe asthma. Parallel immunological and clinical profiling of children participating in a birth cohort study has shown an association between AHR to inhaled histamine and a Th1 phenotype characterized by enhanced IFNγ production by peripheral blood mononuclear cells stimulated with phytohaemagglutinin A (PHA) (Heaton et al., 2005). Furthermore, an increase in IFNγ-expressing inflammatory cells has been found in the airway subepithelial compartment in severe, but not in moderate, asthmatics who have sputum neutrophilia and eosinophilia (Shannon et al., 2008). In mouse models, Th1 cells have been shown to function in a cooperative manner with Th2 cells to mediate severe airway inflammatory responses; however, this has not been a universal finding and other studies have reported that Th1 responses can counteract Th2-mediated asthma, with the conflicting results potentially reflecting differences in mouse models or experimental approaches (Hansen et al., 1999; Yu et al., 2011). There exists, nonetheless, a growing body of evidence generated from mouse models that indicates a potential role for IFNγ-dependent Th1 responses in the pathogenesis of neutrophilic airway inflammation and AHR in severe asthma (Fig. 3). For example, the adoptive transfer of antigen-specific IFNγ-producing Th1 cells plus lipopolysaccharide (LPS) to OVA-challenged mice induces steroid-resistant AHR that is dependent upon TLR4 and MyD88 and inhibited by the depletion of macrophages (Yang et al., 2009). Similarly, BALF neutrophilia that can only be partially suppressed by steroid treatment is induced by the adoptive transfer of Th1 cells together with LPS. In contrast, the adoptive transfer of antigen-specific Th2 cells induces eosinophilic airway inflammation and AHR that is steroid-responsive. Furthermore, cooperative signaling by IFNγ and LPS induces MyD88-dependent, steroid-resistant AHR via a pathway involving IL-27 production by pulmonary macrophages (Li et al., 2010a). These studies demonstrate the role of cooperative signaling by Th1 cells and LPS in mediating steroid-unresponsive AHR.

Mouse studies have also highlighted a cooperative role for Th1 cells and IL-18 in asthma pathogenesis. The adoptive transfer of antigen-specific IFNγ-producing Th1 cells that express the IL-18 receptor α chain, followed by intranasal challenge with OVA and IL-18, induces IFNγ-dependent AHR and severe airway inflammation (Sugimoto et al., 2004). The cells involved in this response are neutrophils, eosinophils and lymphocytes. Interestingly, these IFNγ-producing Th1 cells also express Th2 cytokines, such as IL-9 and IL-13, as well as TNF, GM-CSF and chemokines. Furthermore, co-administration of OVA plus LPS induces Th1 cells to express IFNγ in an IL-18-dependent manner that leads to severe neutrophilic airway inflammation and AHR, which suggests that this pathway is relevant for asthma exacerbations elicited by bacterial infections (Hayashi et al., 2007). Extending further the role of IFNγ and Th1 pathways in severe asthma, the intranasal administration of IFNγ has been shown to induce AHR in the absence of neutrophilia via a pathway that involves the binding of neurokinin A to neurokinin-2 receptors expressed on airway smooth muscle cells (Kobayashi et al., 2012).

An additional important role for IFNγ in the pathogenesis of asthma has been identified using an engraftment model of mast-cell-deficient *Kit*^W-sh/W-sh^ mice (Yu et al., 2011). Expression of the IFNγ receptor by mast cells promotes AHR, neutrophilic and eosinophilic airway inflammation, and airway remodeling, which defines a newly identified role for IFNγ-dependent mast cell activation in the pathogenesis of chronic allergic airway inflammation in mice. Collectively, these studies suggest that therapies targeting IFNγ might be effective in severe asthmatics with Th1-driven immune responses.

### Mouse models of the obesity-related severe asthma phenotype

Studies of human asthmatics have identified a subgroup of asthmatics, mostly female subjects, that is characterized by obesity, late onset (mid-40s) disease and minimal allergy (Wenzel, 2012b). Similarly, AHR is a common feature of mouse models displaying an obese phenotype, such as *ob*/*ob* mice that lack the satiety hormone leptin, *db*/*db* mice that lack the leptin receptor, or *Cpe^fat^* mice that lack carboxypeptidase E, which is involved in the processing of neuropeptides that mediate eating disorders (Shore, 2010). However, administration of leptin to OVA-challenged mice induces increases in AHR and serum IgE, suggesting that a more complex mechanism underlies the increased AHR in obese mice (Shore, 2010; Shore et al., 2005). Systemic administration of adiponectin, an anti-inflammatory adipokine, mediates a decrease in AHR, Th2 cytokines, and BALF eosinophils, neutrophils and lymphocytes (Shore et al., 2006). Similarly, OVA-challenged adiponectin knockout mice have increases in BAL eosinophils and macrophages, although AHR is not modified (Medoff et al., 2009).

### Mouse models of the pauci-granulocytic asthma phenotype

The absence of a predominant inflammatory cell in the sputum of a group of severe asthmatics suggests that the pauci-granulocytic phenotype is mediated by non-immune or non-inflammatory mechanisms. Consistent with this hypothesis, a recent study of asthmatics has shown that methacholine-induced bronchoconstriction induces airway remodeling that is manifested by goblet cell hyperplasia, epithelial cell proliferation and basement membrane thickening, without eosinophilic inflammation (Grainge et al., 2011). Several mouse studies have suggested a role for integrin pathways in the pathogenesis of exaggerated airway narrowing, which could contribute to the pathogenesis of inflammation-independent AHR. For example, the αvβ5 integrin, which is expressed in airway smooth muscle cells, mediates increases in allergen-induced airway smooth muscle thickness independently of airway inflammation (Tatler et al., 2011). Furthermore, contraction agonists induce transforming growth factor β (TGFβ) activation in smooth muscle cells via αvβ5, which suggests that this pathway could induce bronchoconstriction-induced airway remodeling in asthma. Integrin α9β1, which is also expressed in airway smooth muscle tissue, has been shown to attenuate airway smooth muscle contraction and AHR via a mechanism involving the recruitment of the polyamine-catabolizing enzyme spermidine/spermine *N*^1^-acetyltransferase, which leads to the inhibition of the lipid kinase PIP5K1γ (Chen et al., 2012). The enzymatic action of PIP5K1γ is the major source of phosphatidylinositol (4,5)-bisphosphate [PtdIns(4,5)*P*_2_; also known as PIP_2_] in airway smooth muscle. Therefore, inhibition of PIP5K1γ leads to a reduction in PIP_2_, which results in a decrease in inositol (1,4,5)-trisphosphate [Ins(1,4,5)*P*_3_; also known as IP_3_]-dependent calcium (Ca^2+^) release from the sarcoplasmic reticulum of smooth muscle, because PIP_2_ is the key precursor for IP_3_ generation. Thus, this pathway might also represent a new therapeutic target for severe-asthma-associated AHR. Consistent with an important role for integrins in AHR, administration of an integrin-blocking peptide attenuated allergen-induced airway smooth muscle hyperplasia and AHR, but not airway inflammation or fibrosis, in a guinea pig model of allergic asthma (Dekkers et al., 2010). Collectively, these studies suggest that therapeutic strategies targeting integrin pathways might be effective in reducing AHR in severe asthmatics.

Several additional non-inflammatory pathways have been identified that might participate in pauci-granulocytic forms of severe asthma. ADAM33, which was identified as an asthma susceptibility gene in a human genome-wide association study, was initially proposed to have a role in airway remodeling and AHR, based on its expression in airway smooth muscle cells and fibroblasts (Chen et al., 2006; Foley et al., 2007; Van Eerdewegh et al., 2002). However, studies using ADAM33-null mice showed that ADAM33 is not involved in allergen-induced AHR, airway inflammation or mucous cell metaplasia (Chen et al., 2006). Interestingly, TGFβ2 induces shedding of cell surface ADAM33, and high levels of soluble ADAM33 have been found in BALF from asthmatics (Puxeddu et al., 2008). Furthermore, ADAM33 has been shown to possess pro-angiogenic activity, which suggests that soluble ADAM33 might promote angiogenesis in the airway and thereby contribute to airflow obstruction independently of airway inflammation.

Ozone exposure has been shown to acutely induce AHR in mice in the absence of inflammation, via the production of a vasoactive mediator, 20-HETE, which is not suppressed by pre-treatment with dexamethasone (Cooper et al., 2010). This suggests a role for 20-HETE in ozone-induced, neutrophil-independent AHR in mice. Lastly, co-administration of poly-L-lysine, which mimics the cationic protein of inflammatory cells, and OVA leads to exaggerated central airway constriction and airway wall thickening in mice, ultimately leading to death. This synergy between increased smooth muscle shortening and increased airway wall thickness could represent a mechanism underlying the pathophysiology of severe asthma exacerbations (Bates et al., 2008).

## Bench to clinical trials: taking forward new candidates for drug development

An important goal of research using mouse models of severe asthma is to identify currently unknown disease pathways and mechanisms that can serve as targets for the development of novel treatment approaches. This review has highlighted how mouse models have been used to accomplish this. The next step is to assess the efficacy and safety of promising candidates in clinical trials. Clinical studies of severe asthmatics have identified multiple subgroups with distinct clinical or inflammatory phenotypes that reflect different mechanisms of disease pathogenesis. Therefore, new treatments that specifically target immunological pathways, such as Th1, Th2 and Th17 immune responses, or inflammatory phenotypes, such as eosinophilic or neutrophilic predominant asthma, will probably need to be administered to individuals whose disease pathogenesis is mediated by a specific pathway, i.e. a personalized approach is warranted. This strategy has already been demonstrated in a subgroup of severe asthmatics presenting with increased sputum eosinophils: disease exacerbations were significantly reduced by treatment with anti-IL-5 monoclonal antibodies (Haldar et al., 2009; Nair et al., 2009; Pavord et al., 2012). The concept of using anti-IL-5 to treat asthma was first investigated in animal models in which anti-IL-5 antibodies were shown to successfully reduce allergen-induced airway eosinophilia (Chand et al., 1992; Mauser et al., 1993; Van Oosterhout et al., 1993). Although 20 years have transpired since these initial animal studies, the administration of anti-IL-5 monoclonal antibodies to severe asthmatics with an eosinophil-predominant inflammatory phenotype to reduce the frequency of disease exacerbations has progressed to Phase III clinical trials (Pavord et al., 2012). Similarly, therapies aimed at inhibiting the Th2 cytokines IL-4 and IL-13 will probably need to be targeted at severe asthmatics in whom this pathway is active, which has been shown to be ∼50% of mild-to-moderate asthmatics (Woodruff et al., 2009). In a recent clinical trial, the anti-IL-13 monoclonal antibody lebrikizumab improved the prebronchodilator FEV_1_ (forced expiratory volume in 1 second) only in the group of poorly controlled asthmatics with a Th2 inflammatory phenotype (as indicated by elevated serum periostin levels), which represented 50% of the cohort (Corren et al., 2011).

Alternatively, new therapeutic strategies that globally suppress airway inflammation and remodeling and AHR or that reverse steroid unresponsiveness could be widely utilized among all severe asthmatics, without the need to target a specific phenotype. An example of this is provided by pro-resolution lipid mediators, such as lipoxins, protectins and resolvins, which were reported as being able to attenuate disease severity in mouse asthma models (Haworth et al., 2011; Haworth et al., 2008; Levy et al., 2005; Levy et al., 2002; Levy et al., 2007a; Levy et al., 2007b). Lipoxin A4 (LXA_4_) is an eicosanoid product that signals via LXA_4_ receptors to promote the resolution of cytokine-mediated inflammation (Levy et al., 2002). An OVA-challenged mouse model of experimental asthma showed that systemic administration of an LXA_4_ analog blocks AHR, eosinophilic airway inflammation and the production of Th2 cytokines, IL-5 and IL-13. A role for LXA_4_ in human disease has also been indicated: calcium-ionophore-treated whole blood samples from severe asthmatic patients exhibit reduced LXA_4_ production compared with moderate asthmatics (Levy et al., 2005). Recently, both NK cells and type 2 ILCs were shown to express LXA_4_-binding receptors, which mediate increased NK-cell-dependent eosinophil apoptosis and reduced IL-13 release by ILCs (Barnig et al., 2013). These studies demonstrate that impaired LXA_4_ biosynthesis might contribute to the pathogenesis of severe asthma. Furthermore, LXA_4_ stable analogs have been shown to block both allergic airway inflammation and AHR in mouse models, thereby demonstrating their potential utility for the treatment of severe asthma (Levy et al., 2007b). Similarly, protectin D1 is an endogenous lipid derived from docosahexaenoic acid in the lung that attenuates the induction of airway inflammation, mucous cell metaplasia and AHR when administered to OVA-challenged mice. The compound can also promote the resolution of airway inflammation when given as a treatment after aeroallergen challenge (Levy et al., 2007a). Finally, resolvin E1 is an omega-3 fatty acid product that has been shown in mouse models to promote resolution of OVA-induced allergic airway inflammation, mucous cell metaplasia and AHR by attenuating IL-23 and IL-6 production, as well as Th17 cell differentiation and survival (Haworth et al., 2008). Moreover, resolvin-E1-mediated resolution of OVA-induced airway inflammation is dependent upon NK cells that express CMKLR1, a resolvin E1 receptor (Haworth et al., 2011). Collectively, data from mouse models support a role for lipoxins, protectins and resolvins, or their analogs, as potential new treatment approaches for severe asthma.

**Clinical and basic research opportunities**

- To conduct basic studies that further define the mechanisms mediating the pathogenesis of severe asthma, particularly those phenotypes characterized by late onset, obesity, steroid-unresponsiveness or non-inflammatory pathways.
- To conduct clinical studies that assess the effectiveness and safety of therapies targeting specific immune responses (e.g. Th1, Th2 and Th17) or cell types (e.g. eosinophils, neutrophils, iNKT cells, T2M cells, innate lymphoid cells) for the personalized treatment of severe asthma.
- To utilize mouse models to further characterize the pathogenic mechanisms of airway remodeling and airway hyperresponsiveness in severe asthma.
- To develop non-atopic mouse models of severe asthma that facilitate the identification of underlying mechanisms.

## How close are we to solving the clinical puzzle of severe asthma?

Many unanswered questions remain regarding the pathogenesis and treatment of severe asthma. These could be addressed by additional research using mouse models. For example, the majority of mouse models utilize acute allergen challenges in relatively youthful mice and therefore model early-onset disease. Because many clinical asthma phenotypes develop later in life, new mouse models that reflect late-onset disease would be very valuable. Moreover, the development of new models that simulate the pauci-granulocytic severe asthma phenotype and focus on non-inflammatory mechanisms of AHR and airway remodeling could identify therapeutic targets that are relevant to these manifestations of disease. Novel mouse strains with enhanced phenotypic features of severe asthma could be investigated using genetic and genomic approaches, such as linkage analysis and next-generation sequencing, to identify additional candidate genes and polymorphisms in known asthma-associated genes. Mouse models could be used to further define the role of epigenetic mechanisms and non-coding RNAs, such as microRNAs and long noncoding RNAs (lncRNAs). In addition, mouse models could be exploited to further characterize the role of genes and polymorphisms that have been identified in genetic studies of severe asthmatics. Therefore, we believe that the strategy of asking clinically relevant questions in animal models to identify currently unknown mechanistic pathways that might be targeted in the development of new treatments remains an attractive and productive research approach to solve the clinical puzzle of severe asthma.
